# Non-IgE-reactive allergen peptides deteriorate the skin barrier in house dust mite-sensitized atopic dermatitis patients

**DOI:** 10.3389/fcell.2023.1240289

**Published:** 2023-08-22

**Authors:** Karin Pfisterer, Matthias Wielscher, David Samardzic, Pauline Weinzettl, Dorte Symmank, Lisa E. Shaw, Raffaela Campana, Huey-Jy Huang, Matthias Farlik, Christine Bangert, Susanne Vrtala, Rudolf Valenta, Wolfgang Weninger

**Affiliations:** ^1^ Department of Dermatology, Medical University of Vienna, Vienna, Austria; ^2^ Department of Pathophysiology and Allergy Research, Center for Pathophysiology, Infectiology, and Immunology, Medical University of Vienna, Vienna, Austria; ^3^ Karl Landsteiner University of Health Sciences, Krems, Austria

**Keywords:** skin barrier, epithelial barrier disruption, atopic dermatitis, Der p 2, house dust mite, allergy, allergen response in the skin, keratinocytes

## Abstract

Atopic dermatitis (AD) is a chronic inflammatory skin disease characterized by type 2 cytokine-driven skin inflammation and epithelial barrier dysfunction. The latter is believed to allow the increased penetration of chemicals, toxins, and allergens into the skin. House dust mite allergens, particularly Der p 2, are important triggers in sensitized individuals with AD; the precise actions of these allergens in epithelial biology remain, however, incompletely understood. In this study, we compared the effects of the protein allergen Der p 2 and a mix of non-IgE-reactive Der p 2 peptides on skin cells using patch tests in AD patients and healthy participants. We then analyzed mRNA expression profiles of keratinocytes by single-cell RNA-sequencing. We report that existing barrier deficiencies in the non-lesional skin of AD patients allow deep penetration of Der p 2 and its peptides, leading to local microinflammation. Der p 2 protein specifically upregulated genes involved in the innate immune system, stress, and danger signals in suprabasal KC. Der p 2 peptides further downregulated skin barrier genes, in particular the expression of genes involved in cell–matrix and cell–cell adhesion. Peptides also induced genes involved in hyperproliferation and caused disturbances in keratinocyte differentiation. Furthermore, inflammasome-relevant genes and IL18 were overexpressed, while KRT1 was downregulated. Our data suggest that Der p 2 peptides contribute to AD initiation and exacerbation by augmenting hallmark features of AD, such as skin inflammation, barrier disruption, and hyperplasia of keratinocytes.

## 1 Introduction

The human skin shields the body against physical, chemical, and immunological threats, preventing the loss of body fluids and the entry of substances from the environment. The epidermis is the outermost layer of the skin and builds the first line of defense. It consists of keratinocytes (KCs) that are continuously renewed and maintained by proliferating cells in the basal layer (Blanpain and Fuchs, 2009). Basal KCs are located on the basement membrane (BM), a sheet-like extracellular matrix (ECM) that separates the dermis and epidermis. The BM is composed of collagen type IV (Col IV), laminin, and fibronectin. Basal KCs strongly adhere to ECM molecules of the BM through supramolecular cell adhesion complexes called hemidesmosomes that contain α6β4 integrin receptors (Garrod, 1993). These complexes establish connections with keratin intermediate filaments (KRT5 and KRT14) and transmembrane Col XVII (Nahidiazar et al., 2015). Basal KCs also exhibit focal adhesion through α/β1 integrins, linking F-actin fibers to the ECM and allowing cell attachment to the BM (Muroyama and Lechler, 2012).

When differentiating into suprabasal KCs, encompassing cells of the stratum spinosum and granulosum, basal KCs weaken their connection with the BM and migrate to the upper layers of the skin. During this process, differentiating KCs reduce their mitotic activity and increase cell–cell interactions in a calcium-dependent mechanism (Carter et al., 1990). Suprabasal KCs downregulate the expression of KRT5 and KRT14 and instead express KRT1 and KRT10, which are connected to robust intercellular junction complexes called desmosomes that stabilize cells and the epithelium (Garrod, 1993; Candi et al., 2005; Blanpain and Fuchs, 2009). Another stabilizing cell–cell connection in the epidermis is adherens junctions, which connect the actin cytoskeleton to the plasma membrane through cadherins (Candi et al., 2005). Additionally, KCs in the granular layer form tight junctions, which contribute to the formation of a dense skin barrier, limiting the entry of molecules that are larger than 500 Da (Da) (Kabashima et al., 2019; Beck et al., 2022). Tight junctions are intercellular belt-like adhesion complexes that connect to F-actin and consist of occludins, claudins, and junctional adhesion molecules (Mertens et al., 2005). They regulate the paracellular molecule passage and prevent transepidermal water loss (Tsakok et al., 2019; Yazici et al., 2022). The barrier function of the skin is mostly dependent on tight junctions and the stratum corneum, the outermost epidermal layer. The stratum corneum is formed through crosslinking of structural proteins and lipids during the terminal differentiation of KCs (Zeeuwen, 2004). Desmosomes, hemidesmosomes, and focal adhesions further regulate the skin barrier function and must be dynamically controlled during cell proliferation and differentiation (Huber et al., 2023).

Atopic dermatitis (AD) is a chronic or relapsing inflammatory skin disease affecting 10%–20% of people in the Global North (Weidinger and Novak, 2016). AD typically develops in early childhood and can persist into adulthood, manifesting in dry, itchy, and inflamed skin lesions (Weidinger and Novak, 2016). This skin disease is characterized by elevated type 2 cytokines, high allergen-specific IgE (in atopic forms), an imbalance of skin microbiota, and a pronounced disruption of the epidermal barrier. In 20%–40% of patients, the barrier dysfunction is caused by a loss-of-function mutation of filaggrin, which is mainly expressed in granular KCs (Langan et al., 2020). Filaggrin binds keratin filaments and is secreted into the stratum corneum, contributing to the barrier formation along with lipids (Langan et al., 2020). Even in the absence of this mutation, filaggrin expression is often decreased in AD patients through downregulation by type 2 cytokines such as IL-4 and IL-13 (Moosbrugger-Martinz et al., 2022). Type 2 immune responses also weaken the skin barrier by reducing the expression of stratum corneum lipids and genes involved in the formation of tight junctions (Langan et al., 2020; Beck et al., 2022). A dysfunctional epidermal barrier induces stress on KCs, resulting in the secretion of proinflammatory cytokines and chemokines, DAMPs, and alarmins, such as IL-1 family cytokines, KRT6, and KRT16 (Lessard et al., 2013; Leung et al., 2020). Pruritus-induced scratching and an imbalance in the bacterial skin microbiome can also induce alarmins, which, in turn, can provoke type 2-mediated immune responses (Leung et al., 2020). This often results in chronic inflammation through a positive feedback loop.

The factors responsible for initiating the development of AD and triggering relapse after the clearance of inflammation are yet not well understood. However, it has been suggested that a dysfunctional epidermal barrier plays a crucial role by allowing the penetration of chemicals or allergens. House dust mite (HDM) allergens, in particular, can induce skin inflammation in allergic patients with AD, but the exact mechanisms are not fully understood (Kaplan et al., 2012; Serhan et al., 2019). Der p 2 from *Dermatophagoides pteronyssinus* is one of the major air-borne HDM allergens (Huang et al., 2019). It can trigger type I hypersensitivity reactions, resulting in IgE responses, high type 2 cytokine production, and histamine release. HDM allergens can also act as a contact allergen and cause local skin inflammation in sensitized individuals, which has been shown in isolated human cells and mouse models (Liedén et al., 2009; Kaplan et al., 2012; Stremnitzer et al., 2014). In healthy skin, allergens with molecular weights of >500 Da cannot pass through the epidermal barrier due to size constraints (Smith et al., 2017). The HDM allergen Der p 1 is approximately 25 kDa in size and can directly bypass the skin barrier using its intrinsic papain-like proteases to disrupt cell–cell adhesions between KCs (Reithofer and Jahn-Schmid, 2017). This is in contrast to Der p 2 (15 kDa), which has no protease activity but may be fragmented into peptides through highly abundant proteases in HDM feces. Furthermore, the skin of people with AD is believed to be more permissive due to a barrier deficiency and may allow deeper penetration of allergens compared to healthy skin.

To date, there is limited understanding of the mechanisms underlying the transition from a non-lesional skin with a barrier deficiency to acute lesional skin with a pronounced barrier disruption in AD. In this study, we compared the ability of the recombinant HDM allergen Der p 2 and a mix of hypoallergenic Der p 2 peptides, which lack IgE reactivity and do not induce basophil activation (Huang et al., 2019), to induce skin inflammation in both AD patients and healthy participants using single-cell RNA sequencing (scRNA-seq). We report here that the existing barrier deficiency in the non-lesional skin of AD patients allowed deep penetration of both protein and peptide allergens. Der p 2 protein caused local microinflammations, as evidenced by the activation of immune system-relevant genes. Non-IgE-reactive Der p 2 peptides downregulated the expression of KRT1 and upregulated the expression of the inflammasome gene *PYCARD* and the alarmin IL18. Furthermore, Der p 2 peptides significantly disrupted the skin barrier by downregulating the expression of cell–cell and cell–matrix adhesion genes and induced genes associated with hyperproliferation in KCs. Based on our observations, we propose that Der p 2 peptides are involved in disrupting the skin barrier in AD patients sensitized to HDM and thereby contribute to acute disease exacerbations.

## 2 Materials and methods

### 2.1 Study participants and ethics statement

The study was approved by the Ethics Committee of the Medical University of Vienna (#2472/2020) and is in accordance with the Declaration of Helsinki principles. Patients were informed about the study procedures, benefits, and risks and gave their written informed consent. Four patients, who were diagnosed with atopic dermatitis according to the Hanifin–Rajka criteria and with reported allergy to house dust mite (HDM), and four healthy participants without any records of chronic inflammatory skin diseases and HDM sensitization were recruited to participate in this study. All participants were aged between 18 and 80 years (Supplementary Figure S1A). Adult human skin samples were obtained as discarded materials from routine plastic surgery in accordance with the Declaration of Helsinki principles and after approval by the Ethics Committee of the Medical University of Vienna.

### 2.2 Expression and purification of recombinant Der p 2 and synthesis of Der p 2-derived peptides

For the expression and purification of recombinant Der p 2 (Der p 2 rec), the cDNA coding for Der p 2 (GenBank accession number AF276239) was amplified by RT-PCR using mite RNA, as previously described (Chen et al., 2008). PCR products of Der p 2 cDNA contained NdeI and EcoRI sites in the upstream region and an EcoRI site in the downstream region, as well as six His codons, and were subcloned into the plasmid pET-17b expression vector (GenScript, United States). The vector was introduced into ClearColi™ BL21 (DE3) electrocompetent cells (Lucigen, Wisconsin, United States) by electroporation using a MicroPulser Electroporator (program Ec2: 0.2-cm cuvette and 2.5 kV) (Bio-Rad, United States). Expression of Der p 2 rec was induced by adding 1 mM isopropyl-β-thiogalactopyranoside (IPTG) at an OD_600_ of 0.6 for 4 h at 37°C in the LB medium with 100 μg/mL ampicillin. Cells from 500 mL cultured medium were harvested by centrifugation (2,000 g for 20 min at 4°C), and cell pellets were then dissolved in 15 mL lysis buffer (25 mM imidazole, 0.1% Triton X-100, and pH 7.4) by mixing for 20 min at room temperature. Cell lysates were obtained by three consecutive freeze–thaw cycles (−70°C/+50°C). Incubation with 2 μg DNase I for 10 min at room temperature was performed to remove DNA, and thereafter, 100 mM NaCl was added to the lysates. Der p 2 rec was detected in the pellets (inclusion body) after centrifugation (38,900 g for 20 min at 4°C) and solubilized by mixing with a buffer containing 8 M urea, 100 mM NaH_2_PO_4_, and 10 mM Tris-Cl (pH 8) for 3 h at room temperature. After centrifugation (38,900 g for 20 min at 4°C), Der p 2 rec protein in the supernatant was incubated with Ni-NTA resin overnight at 4 °C and bound Der p 2 rec was eluted from Ni-NTA resin affinity columns (QIAGEN, Hilden, GER) using 8 M urea, 100 mM NaH_2_PO_4_, and 10 mM Tris-Cl (pH 4.5). Purified Der p 2 rec was then dialyzed with 10 mM NaH_2_PO_4_ (pH 4.7) to increase solubility and prevent precipitation. Endotoxin of Der p 2 rec was <10 EU/mL determined by LAL assays (Pierce™ LAL Chromogenic Endotoxin Quantitation Kit, Thermo Scientific, United States).

Five overlapping peptides derived from Der p 2 with a length between 31 and 42 amino acids covering the full sequence of Der p 2 were synthesized using a peptide synthesizer (Liberty, CEM Corporation, Kamp-Lintfort, GER) and reconstituted in sterile endotoxin-free water, as previously described (Huang et al., 2019). Der p 2 peptides were defined as hypoallergenic peptides due to the lack of IgE reactivity and the disability to induce basophil activation. Five Der p 2-derived peptides (Der p 2 pep) and Der p 2 rec were filtered using 0.2-μm sterile syringe filters (Thermo Scientific), and the concentration of protein and peptides was determined using the BCA Protein Assay Kit (Pierce, Rockford, Illinois, United States).

### 2.3 Patch test, tissue sampling, and cell isolation

Non-inflamed skin on the upper back of AD patients and healthy participants was tape-stripped 10 times, and 40 μg of Der p 2 rec, and an equimolar mix of Der p 2 pep, saline (0.9% NaCl), and water (all sterile) were applied onto four separate nonwoven fabric spots of adhesive strips for patch tests (Curatest^®^, Lohmann & Rauscher, GER). The adhesive strips were further secured using a water-repellent plaster. Participants were invited back to the clinics 72 h later, and patch test areas were evaluated for inflammation. One 6-mm biopsy was taken from the skin treated with allergen and one from the skin treated with allergen-derived peptides (two biopsies in total per participant), and biopsies were separately stored in sterile, precooled phosphate-buffered saline for a maximum of 20 min until further processing. Skin biopsies were cut into small pieces using a scalpel, and cells were isolated via enzymatic digestion for 1.5 h using the Gentle MACS Whole Skin Dissociation Kit, according to the manufacturer’s protocol (Miltenyi Biotec, GER). Single cells were resuspended in scRNA-seq resuspension buffer (1x PBS +0.04% BSA (w/vol), sterile), and counted using 0.4% trypan blue solution in saline (Corning, United States). Overall, all samples were processed within 3 h from taking the biopsy until processing for next-generation sequencing.

### 2.4 Single-cell RNA sequencing (scRNA-seq)

In total, 30,000 living cells were loaded into a 10x Genomics Chromium Controller for the generation of single-cell droplets. DNA and transcriptome libraries were generated following the 10x Genomics Next GEM Single Cell 5ʹ V2 protocol. Quality control was performed using Qubit (Invitrogen #Q33231) and the TapeStation system (Agilent). Libraries of six samples from three donors were sequenced on two lanes of one NovaSeq SP flow cell using a NovaSeq 6000 system with a read length of 2 × 50 bp, resulting in an average of 100 million reads per sample. Reads were demultiplexed and analyzed using Cell Ranger.

### 2.5 EmptyDrops, doublet removal, and quality control (QC) for scRNA-seq

To differentiate between background noise and cell containing droplets, we used emptyDrops (Lun et al., 2019), which models the ambient RNA background within the dataset and identifies deviations from this background RNA. We applied a false discovery rate cut off of 0.05 to identify cells to be included into further analysis. To eliminate droplets containing more than one cell, we utilized the scran package from Bioconductor (Lun et al., 2016). The doublet score was calculated based on the simulation of thousands of doublet cells by adding together two randomly chosen single-cell profiles. For the doublet score calculation, the cells and the set of randomly generated doublet cells were clustered. Then, for each cell, the number of simulated doublets in its neighborhood was recorded and used as the input for score calculations. We used 200 nearest neighbors for each cell and applied a threshold of doublet score >4 to identify doublets in each dataset separately. The doublet score was defined as log10 of the ratio between simulated doublet cells and the total number of neighbors taken into consideration for each cell. Following the quality control process, we obtained a count of more than 3,000 cells for each sample (total of 50,000 cells), with approximately 1,690 genes per cell and 169 UMI per gene for the final analysis (Supplementary Table S1).

### 2.6 Analysis of scRNA-seq data

After performing individual quality control of the samples, the raw read counts from all datasets were merged into one count matrix. To conduct principal component analysis and differential gene expression analysis, we used Pearson residuals that were derived from a generalized negative binomial model of UMI counts, which is implemented in the R package sctransform (Hafemeister and Satija, 2019) using Seurat (Satija et al., 2015). In addition, we adjusted the regression model for sequencing depth, mitochondrial RNA content, and experimental batch effects. We removed cells with the mitochondrial RNA content above 15%. Furthermore, batch correction across individual datasets was performed using the Harmony algorithm (Korsunsky et al., 2019). Harmony uses batch information provided by the user and then utilizes fuzzy clustering to assign cells to multiple clusters in a manner that maximized batch diversity within each cluster. Correction factors for each cell were obtained by calculating global and batch-specific centroids for each cluster, and the procedure was repeated until convergence of global and batch-specific centroids. tSNE analysis of whole skin samples was performed using PCs 1–15. Clusters were assigned based on the nearest-neighbor-based clustering analysis. We observed a saturation of possible generated cluster at a resolution of 0.7, which was then chosen for further analysis. To assign clusters to known cell types in the skin (Tan et al., 2013; Shih et al., 2017; Cheng et al., 2018; Haensel et al., 2020; Wang et al., 2020; Reynolds et al., 2021; Polkoff et al., 2022), we visualized known cell type-specific markers within the tSNE plot. We also performed a cluster-specific regression analysis, providing us with a list of specific markers for each cluster. Out of approximately 50,000 analyzed cells, we identified 18 skin cell clusters, among which we found 4 KC clusters that were extracted for further analysis (data not shown). Extracted KC cells were re-analyzed including filtering out low expressed genes, calculating Pearson residuals from count data (i.e., data normalization), PC calculation, and clustering. Due to less variability in the keratinocyte dataset, it was sufficient to include PC 1–5 into cluster analysis and tSNE projections. We performed cluster assignment at a resolution of 0.2 to identify eight separate KC clusters. Based on the visual inspection of gene expression of cluster-specific markers (Supplementary Figure S1C) and analysis of gene lists from the Wilcoxon rank-sum test specific for each KC cluster, we defined the following KC cell clusters: basal 1, basal 2, proliferating/mitotic, granular 1, granular 2, spinous, hair follicle, and sebaceous gland KC clusters. To see whether or not the numbers of cells per cluster differ between AD patients and healthy controls, we counted the cells per cluster separately for AD and healthy control samples and performed a chi-squared test to evaluate whether cell numbers differed significantly.

### 2.7 Trajectory analysis

Trajectory analysis was performed using scVelo (La Manno et al., 2018). The analysis framework is based on the abundance of unspliced and mature (spliced) mRNA. This method assumes that differentiation takes place on a timescale similar to the typical half-life of mRNA. The ratio of spliced and unspliced mRNAs in each cell is used to model the progression of cell states. Thus, the arrows in the trajectory analysis plot (Figure 2) indicate a decrease in unspliced mRNA and/or an increase in spliced mRNA, as shown in Supplementary Tables S11–S13.

### 2.8 Pathway analysis

Downregulated genes in AD versus healthy skin (Supplementary Table S6) and differentially regulated genes in AD_pep versus AD_rec (Supplementary Table S10) were used to identify gene sets enriched in GO biological process pathways in the STRING database (Snel et al., 2000). Furthermore, pathway analyses of all genes differentially regulated in AD_pep versus AD_rec were analyzed using the fold change values for gene expression data in the Reactome database (Fabregat et al., 2015). Genes driving RNA velocity trajectories for the merged dataset comprising AD_rec, AD_pep, H_rec, and H_pep were analyzed using Enrichr (Chen et al., 2013). Identified pathway genes were used for bubble plots and to visualize the percentage of cells expressing involved genes in AD, H, AD_rec, or AD_pep using Prism (GraphPad Software, United States).

### 2.9 Intercellular communication network analysis

The probability of cell–cell communications via soluble cytokines and ligands was analyzed using CellChat (Jin et al., 2021). Briefly, gene expression data from our scRNA-seq dataset were integrated with the published data on signaling ligands and receptors to model the probability of cell–cell communications. We modeled the intercellular communication probability for keratinocyte and fibroblast subsets of the merged AD or H datasets for CCL and CXCL signaling pathways to investigate signals from the epidermis to the dermis and *vice versa* using hierarchy plots.

### 2.10 *In vitro* barrier disruption assay

KCs were isolated from human skin after 16–18 h incubation with 2.4 U/mL dispase II (Roche Diagnostics) in PBS at 4°C. The epidermis was separated from the dermis, and single cells were prepared using 0.5% trypsin-EDTA (Invitrogen). KCs were expanded using a serum-free Ca^2+^ low growth medium (PromoCell). For imaging, KCs were seeded into eight-well imaging dishes (IBIDI) and differentiated using 2 mM Ca^2+^. KC sheets were incubated with 5 μg/mL sterile Der p 2 rec or Der p 2 pep (or equal volumes of the solvents PBS or H_2_O) for 24 h and thereafter washed with 1x PBS, fixed with 4% PFA, permeabilized with 0.2% Triton X-100, blocked for 1 h with 5% BSA–PBS, incubated for 2 h with primary Ab, (monoclonal mouse anti-human claudin 1, clone 2H10D10, Invitrogen), and detected with an anti-mouse secondary Ab conjugated to AF488 (Invitrogen). Cell nuclei were stained with DAPI, and cells were imaged using a confocal microscope (FV3000 from Olympus). Images were analyzed using Fiji (Schindelin et al., 2012), and intensities were expressed as arbitrary units (mean fluorescence intensity) normalized to the respective control.

### 2.11 ELISA

IL18 was detected and measured in the serum from HDM-sensitized AD patients or non-sensitized healthy participants using an ELISA Kit (Cloud-Clone Corp.), according to the manufacturer’s instructions.

### 2.12 Statistical analysis

Statistical tests were performed using Prism 9 (GraphPad). Unless stated otherwise, statistical differences were evaluated using the Student’s *t*-test. Data are expressed as mean ± SD, and a *p*-value < 0.05 was considered significant.

## 3 Results

### 3.1 KC subsets in the human skin

**FIGURE 1:**
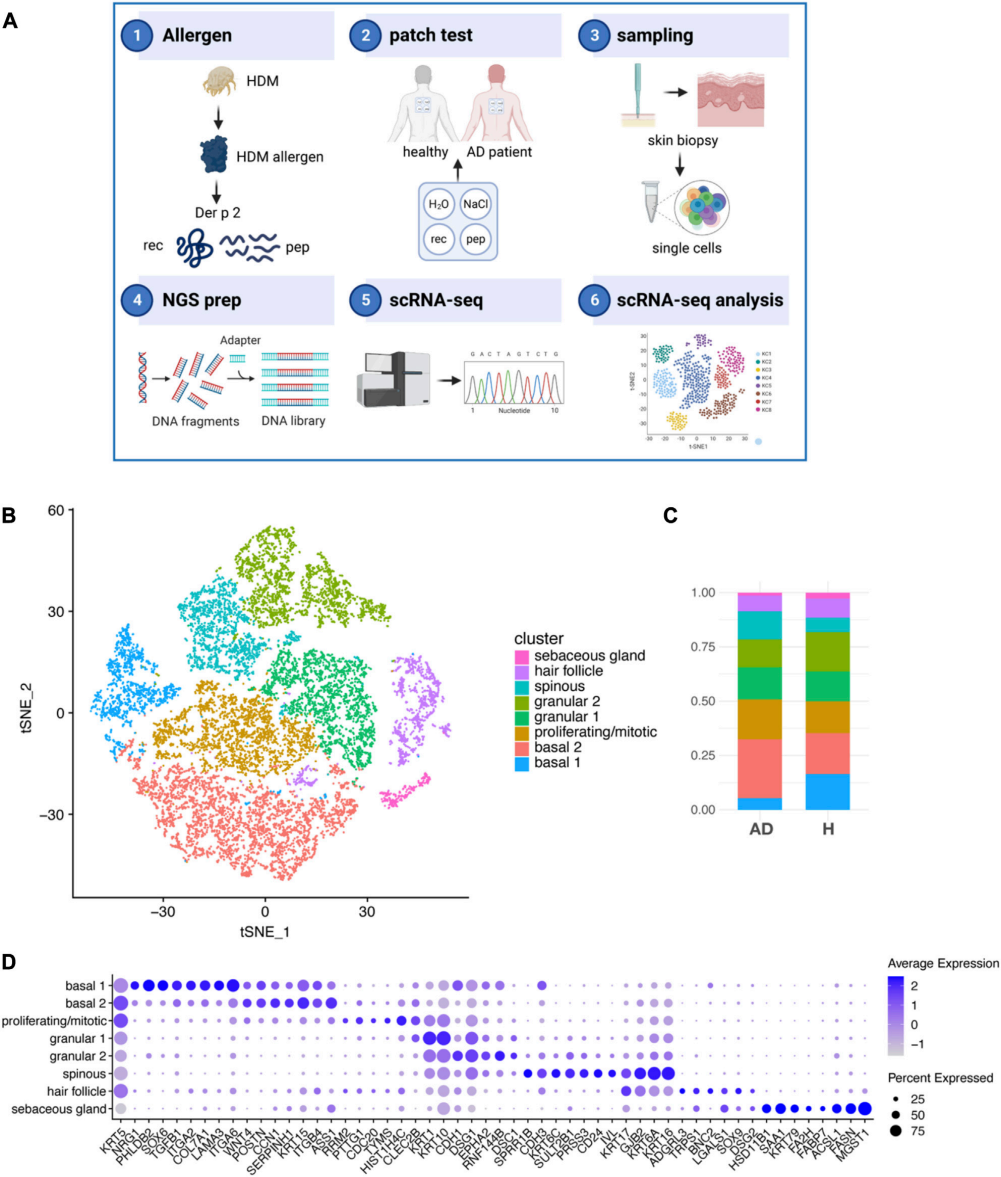
Keratinocyte subsets from AD and H after exposure to HDM allergens. **(A)** Schematic outline of the study strategy: 1) the recombinant HDM allergen Der p 2 (Der p 2 rec), a mix of five hypoallergenic Der p 2 peptides (Der p 2 pep) and negative controls were 2) applied onto the non-lesional back skin of four non-sensitized participants without AD (healthy) and four HDM sensitized participants with AD using patch tests. 3) Biopsies of Der p 2 rec- and pep-treated skin were taken after 72 h, and single cells were produced by the digestion of whole skin tissue. 4) NGS libraries were prepared and 5) sequenced using scRNA-seq technology. 6) Sequencing data from 16 skin samples were processed and KC analyzed. **(B)** tSNE plot showing eight different KC clusters from the merged dataset comprising 16 skin biopsies (4x AD and 4x H, each 1x rec and 1x pep; summing up to 52,975 KC in total). **(C)** Bar charts showing the relative abundance of KC subsets within AD and H samples (*n* = 8 biopsies for AD and H). **(D)** Bubble plot shows the expression of cluster specific markers (*x*-axis) for KC subsets (*y*-axis). The average expression is represented by blue values (low expression, light blue; high expression, dark blue). The percentage of cells expressing the respective marker is represented by the size of the circles. AD, atopic dermatitis; H, healthy; HDM, house dust mite; KC, keratinocyte; scRNA-seq, single-cell RNA sequencing; rec, recombinant; pep, peptide; tSNE, t-distributed stochastic neighbor embedding.

Allergic diseases and chronic inflammatory skin pathologies are increasing, but whether skin flares and epithelial barrier impairment can be directly triggered by aeroallergens is still elusive. Here, we investigated the effect of the house dust mite allergen Der p 2 on the skin of AD patients sensitized to house dust mite (HDM) and healthy non-sensitized individuals (Supplementary Figure S1A). Recombinant Der p 2 allergens (Der p 2 rec) or a mix of hypoallergenic Der p 2 peptides (Der p 2 pep) were applied onto the non-inflamed back skin of HDM-allergic AD patients or non-allergic healthy individuals (Figure 1A; Supplementary Figure S1B). Biopsies for rec and pep-exposed skin were taken after 72 h, and KCs were analyzed by single-cell RNA sequencing (scRNA-seq).

We identified eight KC clusters (Figure 1B; Supplementary Figure S1C) in our merged dataset with a unique expression pattern of specific genes (Supplementary Tables S2–S5 and Figure 1D). Cells of all KC clusters were found in the skin of AD patients (AD) and healthy participants (H) 72 h after Der p 2 protein and peptide exposure at slightly varying amounts (Figure 1B). KCs from the clusters basal 1, granular 2, and sebaceous gland were reduced, whereas KCs from the clusters basal 2, proliferating/mitotic, and spinous were increased in AD compared to H skin. The basal 1 cluster was defined by high expression of hemidesmosome genes, such as alpha 6 and beta 4 integrins (ITGA6 and ITGB4), which bind to laminin, and collagen type 17 (COL17A1, Figure 1D). Both basal 1 and basal 2 expressed high amounts of the intermediate filaments keratin KRT5 and KRT15 and the integrins ITGA2 and ITGB1, which localize to focal adhesions and bind to collagen but have also been shown to be important for cell–cell interactions (Carter et al., 1990). KCs of the basal 2 cluster expressed high amounts of ASS1, which is enriched between rete ridges and may play a role in regulating dermal papilla integrity (Wang et al., 2020). The proliferating/mitotic KC cluster was defined by elevated expression of cell cycle regulator genes, such as *RRM2*, *PTTG1*, *CDC20*, *TYMS*, and *HIST1H4C* (Wang et al., 2020). *KRT1* gene expression was already detectable in mitotic KCs and was most abundant in granular KCs (clusters granular 1 and 2), as described previously (Cohen et al., 2022). The granular KC clusters granular 1 and granular 2 and spinous KC specifically expressed KRT10, CDH1, EPHA2, and the desmosome-specific genes desmoglein (DSG1) and desmocollin (DSC1) at various levels (Figure 1D) (Wang et al., 2020). Spinous KC expressed high levels of the differentiation marker cornifin-B (SPRR1B), CD24, involucrin (IVL), and mesotrypsin (PRSS3) (Tan et al., 2013; Miyai et al., 2014; Cheng et al., 2018; Wang et al., 2020). The spinous cluster also expressed KRT6A, KRT6C, and KRT16, which have been associated with inflammatory skin diseases and barrier dysfunction (Lessard et al., 2013; Cheng et al., 2018; Leung et al., 2020). Both spinous and hair follicle KCs expressed the gap junction genes *GJB2* and *KRT17*. KCs in the hair follicle cluster expressed the stem cell marker SOX9, galectin 1 (LGALS1), basonuclin 2 (BNC2), and latrophilin 3 (ADGRL3), and KCs from the sebaceous gland cluster expressed KRT79, fatty acid-binding protein FABP7, fatty acid hydroxylase FA2H, fatty acid synthase FASN, and the sebocyte marker MGST1 (Shih et al., 2017; Cheng et al., 2018). Taken together, we found all major KC subsets that have been previously identified in the adult human skin (Cheng et al., 2018; Reynolds et al., 2021).

### 3.2 Trajectory and pathway analyses identify AD-specific perturbations in KCs

**FIGURE 2:**
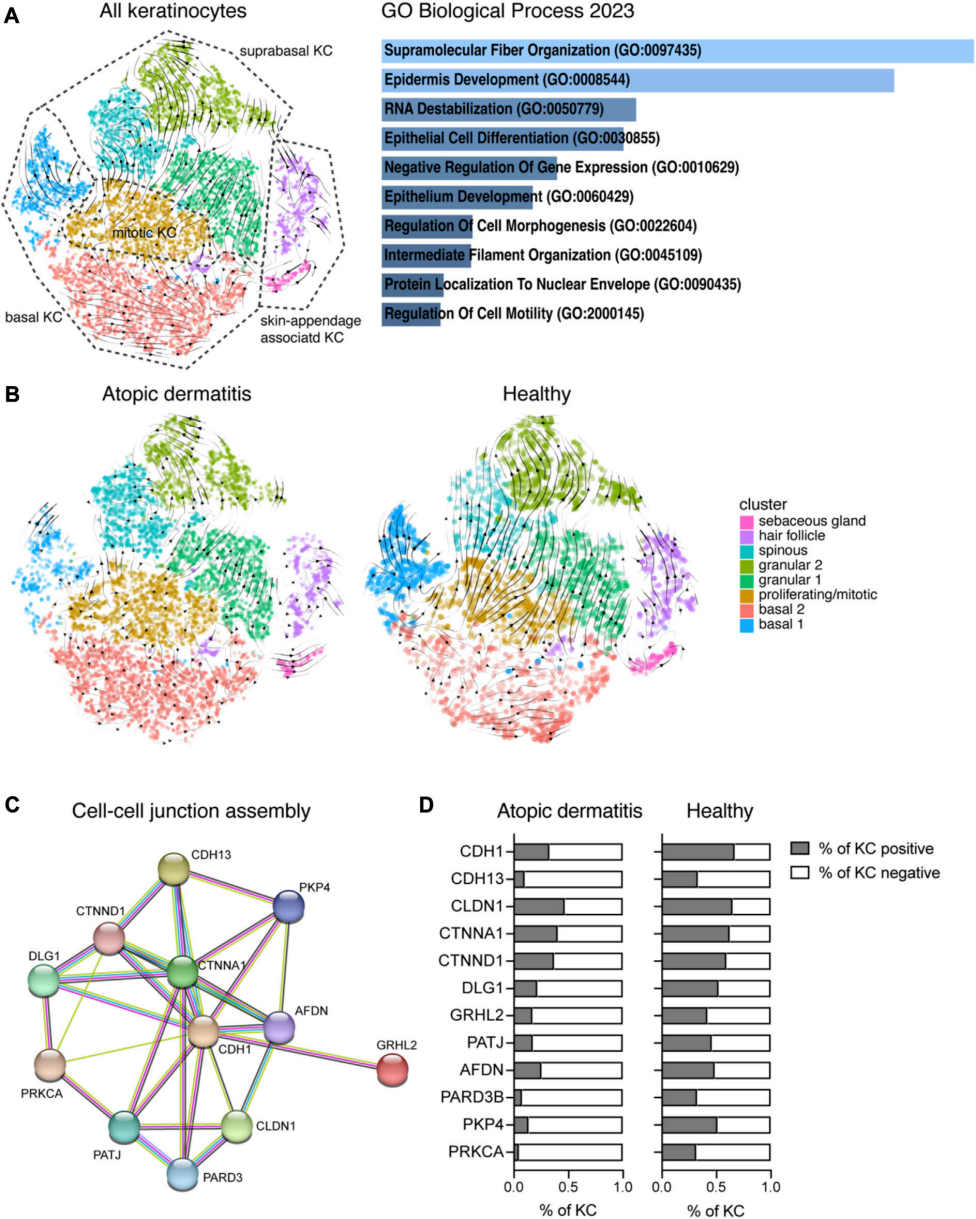
Analysis of the ratio between unspliced and spliced mRNAs reveals AD-specific disturbances in KC differentiation. **(A)** Left: RNA velocity vectors were projected on the tSNE plot from Figure 1B showing KC clusters of the whole dataset (cells from 16 biopsies, AD and H). Right: 140 genes that were driving RNA dynamics were analyzed using Enrichr (Chen et al., 2013). The bar graph shows gene set enrichments in the GO biological process 2023 database (sorted by *p*-value ranking). **(B)** RNA velocity vector projections are shown for KC from treated AD (left) and H (right) skin. **(C)** Functional enrichment analysis identified cell–cell junction perturbations in AD. Downregulated genes in AD were analyzed using the STRING network database The network view of gene names (circles) and their predicted associations (lines) is shown. **(D)** Percentage of KC from AD and H skin expressing genes listed in **(C)** (eight biopsies per group). AD, atopic dermatitis; H, healthy; KC, keratinocyte; tSNE, t-distributed stochastic neighbor embedding.

To understand the relationship between different KC clusters, we analyzed KC data using the trajectory analysis pipeline scVelo. This tool identifies cell differentiation paths and cell states by analyzing the ratio of unspliced to spliced mRNAs in each cell for a gene transcript (Supplementary Tables S11–S13). When all KCs were used for analysis (AD and H; rec and pep), we found that basal and suprabasal KCs originate from the mitotic cell cluster, and hair follicle and sebaceous gland KCs (skin appendage-associated KCs) have their own progenitor cells (Figure 2A). The four major pathways that were identified as drivers of the trajectory analysis were “supramolecular fiber organization,” “epidermis development,” “RNA destabilization,” and “epithelial cell differentiation,” suggesting that ECM organization, keratinocyte differentiation, and epidermis development are the main pathways associated with RNA dynamics in the whole dataset comprising AD and H samples. When we analyzed KC data from AD and healthy participants separately, we found that trajectories from healthy KC (Figure 2B, right panel) showed again a clear separation between basal and suprabasal KCs. Data from healthy skin highlighted a clear dependency between cell division and KC differentiation and a fluid transition between spinous and granular KCs. A closer look on AD-derived KCs (Figure 2B, left panel) revealed highly disordered trajectories. Mitotic cells were again the origin of basal and suprabasal KCs, but there was higher entropy within different clusters, in particular for the KC clusters basal 1 and basal 2 and the spinous KC cluster. Interestingly, KRT5 and KRT14 expression levels, which is highest in basal KCs in healthy skin, were not efficiently downregulated in differentiating cells in AD patients, further suggesting a disturbance in KC differentiation pathways in AD skin (Supplementary Figure S2A). Suprabasal KCs comprising clusters granular 1 and granular 2 and spinous KC maintained high expression levels of KRT5/14. Our data confirm disturbances in KC cell differentiation pathways in AD skin, which contributes to the disease-specific weakened skin barrier function.

To further understand preexisting perturbations in AD skin, we compared the gene expression profile of AD with healthy skin (pooled data from rec and pep for each group, Supplementary Table S6; see also Supplementary Tables S7, S8 for individual analysis of separate KC clusters). We identified over 2,000 significant genes that were differentially regulated between AD and H (Supplementary Table S6). As we aimed to identify pathways involved in AD barrier dysfunction, we further analyzed genes that were downregulated in AD using the STRING database. We identified functional enrichments in GO biological process pathways, such as the “cell–cell junction assembly,” (Figure 2C) “tight junctions,” and “cell–cell adhesion mediated by cadherin” (Supplementary Figure S1D), which are essential components of a functional epidermal barrier. When we had a closer look at genes that are specific for the cell–cell junction assembly (Figure 2C), we identified downregulated gene expression for adherens junction markers, such as E-cadherin (CDH1), alpha- and delta-catenin (CTNNA1 and CTNND1), afadin (AFDN), DLG1, GRHL2, and plakoglobin 4 (PKP4). T-cadherin (CDH13) was previously described to be expressed in basal KCs, and it has been suggested that it plays a role in cell–matrix adhesion (Zhou et al., 2002; Mukoyama et al., 2007). Furthermore, we identified downregulated genes that are involved in the tight junction assembly, such as claudin 1 (CLDN1), PATJ, and PARD3 (Figure 2C; Supplementary Figure S1D). Reduced protein kinase C alpha (PRKCA) expression may downregulate both adherens and the tight junction assembly. Gene expression for cell–cell junction genes was not only downregulated, but also the percentage of KCs expressing those genes was reduced in AD compared to H skin (Figure 2D). Furthermore, we analyzed cell–cluster-specific expression of the tight junction genes *CLDN1*, *PARD3*, and *TIAM1* (Mertens et al., 2005) in KCs from AD patients treated with recombinant Der p 2 (AD_rec) and Der p 2 peptides (AD_pep) and compared it with KCs from healthy individuals that were treated with the same allergens and allergen peptides (H_rec and H_pep) (Supplementary Figure S2B). Tight junction gene expression was highest in granular, spinous, and hair follicle KCs from healthy skin and clearly downregulated in AD patients, independent of allergen exposure. Our data reveal preexisting epithelial barrier damage in AD patients in suprabasal KCs, which is mainly driven by downregulation of cell–cell adhesion genes and confirms previously published data for chronic type 2-driven inflammation (Akdis, 2021).

### 3.3 Der p 2 induces a pro-inflammatory gene expression signature in granular and spinous KCs

**FIGURE 3:**
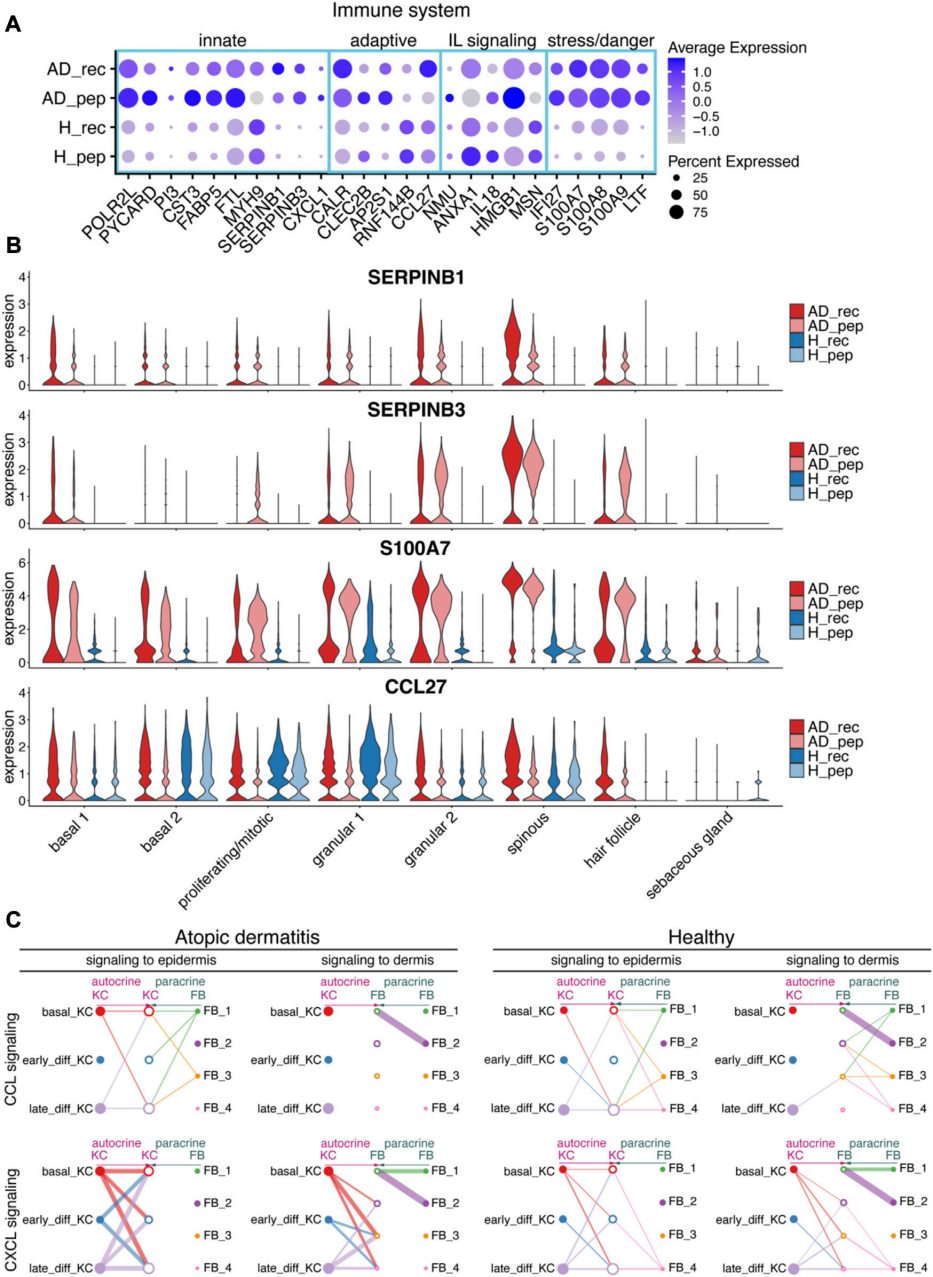
House dust mite allergen Der p 2 initiates inflammatory pathways in KC. **(A)** Bubble plot depicting the expression of immune system-relevant genes in KC from skin exposed to Der p 2 protein (AD_rec, H_rec) and Der p 2 peptides (AD_pep, H_pep). Blue rectangles highlight genes relevant for the innate IS, the adaptive IS, interleukin (IL), and stress/danger signaling. **(B)** Violin plots show the average gene expression in KC clusters from AD_rec (dark pink), AD_pep (light pink), H_rec (dark blue), and H_pep (light blue) skin samples (*n* = 4). **(C)** Analysis of signaling crosstalk via soluble and membrane-bound factors in KC and FB. Hierarchy plots with the signal source plotted left for autocrine signaling (pink) and right for paracrine signaling (green) are shown. The receiving cell subsets (signal target) were plotted in the middle (left plot: signaling to epidermis; right plot: signaling to dermis). The plots illustrate the probability of cell–cell communications in AD and H for CCL (top panel) and CXCL signaling pathways (lower panel). Thick lines represent high probability of cell–cell interactions. AD, atopic dermatitis; H, healthy; FB, fibroblast; IL, interleukin; IS, immune system; KC, keratinocyte; rec, recombinant; pep, peptide.

We then wanted to investigate the potential of HDM allergens and allergen peptides to induce the expression of inflammation markers in AD and H skin (Figure 3A). Analysis of relevant immune system-related genes in KC from AD_rec, AD_pep, H_rec, and H_pep revealed an upregulation of pro-inflammatory markers that can be assigned to the innate immune system (*POLR2L*, *PYCARD*, *PI3*, *CST3*, *FABP5*, *FLT*, *SERPINB1*, *SERPINB3*, and *CXCL1*) in AD_rec and AD_pep, compared to H_rec and H_pep. Furthermore, markers reported to be relevant for adaptive immune responses (CALR, CLEC2B, AP2S1, and CCL27) and genes encoding for interleukin signaling molecules (*NMU*, *IL18*, and *HMGV1*) showed increased expression, and a high number of KCs from Der p 2 rec- and Der p 2 pep-treated AD skin were expressing these genes. Remarkably, there was also a tendency for increased IL18 levels in the serum of AD patients (Supplementary Figure S3E). Markers that are relevant for stress response and danger signaling, such as the alarmins S100A7, S100A8, and S100A9, the antimicrobial peptide lactotransferrin (LTF), and the interferon inducible protein (IFI27) were upregulated in KCs from AD patient skin treated with either Der p 2 rec or Der p 2 pep. In contrast, expression of the actomyosin stabilizing non-muscle myosin IIA (MYH9) and moesin (MSN), and the anti-inflammatory gene *ANXA1* were downregulated by Der p 2 rec and pep treatment (Figure 3A). Some of the genes with increased expression levels have been reported to be specifically upregulated in lesional skin in AD. For instance, the serine protease inhibitor SERPINB3 is upregulated in AD patients, where its expression correlates with skin inflammation and returns to baseline levels in non-inflamed skin upon treatment (Kawashima et al., 2000; Mitsuishi et al., 2005). Interestingly, despite only mild inflammation after patch testing (Supplementary Figure S1B), SERPIN levels were strongly induced in KC upon Der p 2 rec and pep exposure in AD patients (Figure 3A). Further analysis of SERPINB1 and SERPINB3 in KC clusters revealed that SERPIN was highly upregulated in both AD_rec and AD_pep (Figure 3B), but the fully functional protein Der p 2 rec induced the strongest response in KCs from AD patients, particularly in suprabasal KC (Figure 3B). This suggests that Der p 2 rec induced sub-clinical inflammation, which is called microinflammation in AD skin (Akdis, 2021). It has been shown in an AD mouse model that SERPINB3 regulates the epidermal barrier function, and its overexpression is accompanied by increased expression of S100 proteins (Sivaprasad et al., 2015). Especially, the onset of acute skin lesions in AD is associated with elevated S100A7, S100A8, and S100A9 levels in humans (Gittler et al., 2012). We found that S100A7 expression was highly upregulated in all KC clusters, except sebaceous glands (Figure 3B). S100A7 expression was highest in granular and spinous KC clusters of AD patients upon exposure to Der p 2 rec. Moreover, genes that are relevant for adaptive immune responses appeared to be specifically induced by recombinant Der p 2. CCL27, for instance, acts as a chemotactic signal for T cells, enabling KCs to recruit T cells into the skin upon an allergen encounter. CCl27 expression was upregulated in KCs of the spinous and granular 2 clusters in AD patients through the recombinant Der p 2 protein and remained lower in AD_pep, H_rec, and H_pep samples, reinforcing the idea that the Der p 2 protein can efficiently induce a pro-inflammatory environment in suprabasal KCs.

KCs are one of the first cells that encounter environmental allergens and respond quickly by secreting cytokines that may distribute into deeper layers of the skin. To investigate transcriptional changes in the dermis in response to epidermis-derived signals and *vice versa*, we analyzed CCL and CXCL signaling interaction pathways between KC and fibroblasts (FB) using CellChat (Jin et al., 2021). Interestingly, we found that in AD, FB did not respond to KC-derived CCL signals with altered gene expression, whereas FB from healthy skin responded to signals originating from late differentiating KCs (Figure 3C, upper panel). In contrast, KC-derived signals assigned to CXCL signaling pathways could be associated with gene expression responses in FB clusters 2, 3, and 4 with a high probability for AD (Figure 3C, lower panel). Interestingly, there was no association of KC responses to CXCL signaling derived from FB in AD, whereas KC received signals from FB cluster 4 in healthy skin (Figure 3C, lower panel). This suggests that CCL and CXCL signaling crosstalk pathways between KC and FB may be disrupted in AD.

### 3.4 Der p 2-derived peptides downregulate cell–cell and cell–matrix adhesion genes in AD patients

**FIGURE 4:**
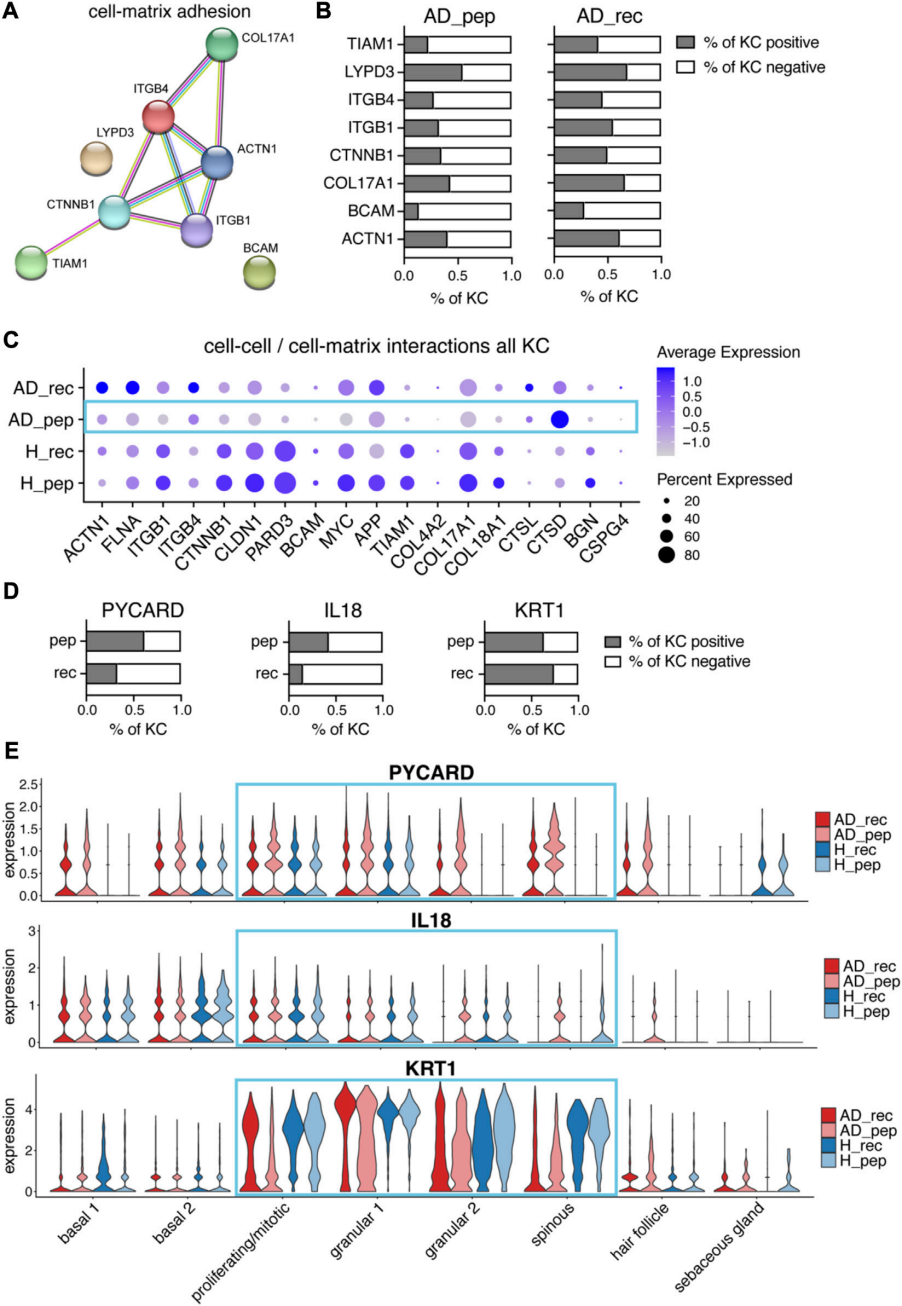
Der p 2 peptides downregulate cell–matrix adhesion genes and upregulate alarmin IL18 in AD. **(A)** Differential gene expression analysis of AD_pep and AD_rec revealed the downregulation of cell–matrix adhesion genes by Der p 2 peptides in KC. The STRING network and predicted functional associations of cell–matrix adhesion genes (*n* = 4) are shown. **(B)** Percentage of KC-expressing genes **(A)** from AD_pep and AD_rec skin (four biopsies per group). **(C)** Bubble plot showing the expression of cell–cell and cell–matrix interaction genes in KC from skin exposed to Der p 2 protein (AD_rec and H_rec) and Der p 2 peptides (AD_pep and H_pep). **(D)** Percentage of KC expressing *PYCARD*, *IL18*, and *KRT1* from AD skin exposed to Der p 2 peptides (pep) and recombinant protein (rec). **(E)** Violin plots depicting the average gene expression of PYCARD (upper plot), IL18 (middle plot), and KRT1 (bottom plot) in KC clusters from AD_rec (dark pink), AD_pep (light pink), H_rec (dark blue), and H_pep (light blue) skin samples (*n* = 4). AD, atopic dermatitis; H, healthy; KC, keratinocyte; KRT, keratin; rec, recombinant; pep, peptide.

Our next aim was to understand the differences between the effects of Der p 2 rec and Der p 2 pep on the skin of AD patients and healthy participants. We did not observe any differential gene expression for the comparison of H_pep versus H_rec, suggesting that recombinant Der p 2 protein and Der p 2 peptides did not induce any significant changes in healthy skin. The global comparison of gene expression data from AD_rec versus AD_pep revealed 19 differentially expressed genes and an enrichment for genes belonging to cell cycle and mitosis pathways (Supplementary Tables S9, S14). In contrast, when we calculated average log2 fold changes between AD_pep and AD_rec for each KC cluster separately, we identified in total 98 significantly down- and 197 significantly upregulated genes (Supplementary Table S10). Downregulated genes were imported into the STRING database and lead to the identification of genes enriched in the GO pathway “cell–matrix adhesion,” such as TIAM1, LYPD3, β4 integrin (ITGB4), β1 integrin (ITGB1), beta-catenin (CTNNB1), COL17A1, BCAM, and alpha-actinin-1 (ACTN1). (Figure 4A). When we had a closer look at the percentage of KC expressing cell–matrix adhesion genes, we saw a reduction in the percentage of KC expressing these marker genes in AD_pep compared to AD_rec (Figure 4B). TIAM1 (T-lymphoma invasion and metastasis) is a Rac-specific guanine nucleotide exchange factor and has been shown to control tight junction biogenesis in KC, thereby controlling barrier formation (Mertens et al., 2005). Alpha actinin-1 (ACTN1) is an F-actin crosslinking protein, which connects F-actin fibers to focal adhesions and hemidesmosomes through interaction with integrins and Col XVII and thereby promotes matrix adhesion (Carter et al., 1990; Hamill et al., 2015). Both BCAM (laminin α5 receptor) and LYPD3 (Ly6/PLAUR domain-containing protein 3) bind laminin in the BM, whereas beta-catenin (CTNNB1) indirectly regulates cell–matrix adhesions by controlling the hemidesmosome assembly through WNT signaling (Kosumi et al., 2022). In addition, the expression of known cell–cell adhesion and ECM organization genes was even further downregulated upon AD skin exposure to Der p 2 pep compared to Der p 2 rec (Supplementary Figures S3A,B). To investigate whether Der p 2 rec or Der p 2 pep could reduce cell–cell adhesion molecules *in vitro*, isolated primary KC were differentiated to form tight junctions incubated with Der p 2 rec or Der p 2 pep, and claudin 1 (CLDN1) expression was analyzed by confocal microcopy (Supplementary Figure S3C). Interestingly, we found that Der p 2 pep significantly reduced CLDN1 protein expression after 24 h of incubation (Supplementary Figure S3D), suggesting a potential direct effect of Der p 2 peptides on keratinocytes.

For a more comprehensive observation at cell–cell and cell–matrix adhesion molecules, we included additional pathway-specific markers into our analysis and compared the average expression and percentage of cells expressing the respective genes for AD_rec, AD_pep, H_rec, and H_pep (Figure 4C). The majority of cell–matrix and cell–cell adhesion genes and genes encoding for ECM organizational proteins (see also Supplementary Figure S3B) was downregulated in KC from the skin of AD patients exposed to Der p 2 pep (AD_pep) compared to AD_rec with the exception of the upregulation of cathepsin L (CTSL) and cathepsin D (CTSD) expression (Figure 4C, blue rectangle). Our data suggest an IgE-independent downregulation of cell–cell and cell–matrix gene expression in the KCs of AD skin, identifying a yet unknown effect of Der p 2 peptides on the skin barrier function.

### 3.5 Der p 2-derived peptides upregulate IL18 and downregulate KRT1 gene expression in AD patients

Cathepsins are proteases, which are considered to be involved in various biological processes such as proenzyme and enzyme activation, tissue remodeling, and matrix remodeling (Zeeuwen, 2004). In particular, lysosomal cathepsins have been shown to activate NLRP3 inflammasomes, which are involved in the recognition of pathogen-associated molecular patterns (PAMPs) and danger-associated molecular patterns (DAMPs) (Li et al., 2021). This prompted us to further investigate inflammasome-related genes that may be involved in danger sensing in the human skin. When we plotted the percentages of KC expressing PYCARD, we found higher amounts of KC expressing this NLRP3-inflammasome adapter protein, which is involved in recruiting the protease caspase-1, in AD_pep compared to AD_rec (Figure 4D, left plot). Interestingly, all KC subsets expressed high levels of PYCARD in AD_pep, except sebaceous gland KCs (Figure 4E, upper plot). In addition, the percentage of KCs expressing the alarmin IL18, a substrate of caspase-1, was increased in KC from the skin of AD patients exposed to Der p 2 pep (Figures 4D and E, middle plots). The upregulation of NLRP3 inflammasome and IL18 gene expression suggests the activation of PAMP or DAMP receptors by Der p 2 pep, and enhanced cathepsin expression may further boost NLRP3 inflammasome activity. Pro-IL18 is processed by activated inflammasomes into its biologically active form. A mouse study has shown that downregulation of KRT1 can further upregulate IL18 expression and diminish the skin barrier function (Roth et al., 2012). In accordance with this study, KRT1 expression was downregulated in KCs from AD_pep (Supplementary Figures S4C, E), and we found elevated IL18 protein levels in the serum from AD patients, although not significant (Supplementary Figure S3E). However, we only identified a minor reduction in the percentage of KC expressing KRT1 (Figure 4D, right plot), suggesting that KRT1 expression was decreased specifically in KRT1-expressing KC subsets. When observing different KC clusters, we identified the Der p 2 pep-specific downregulation of KRT1 expression in proliferating/mitotic, granular, and spinous KCs (Figure 4E, bottom plot). In addition to PYCARD, IL18, and CTSD, we found several other leukocyte-related activation genes to be upregulated in KCs from AD_pep compared to AD_rec such as S100A7-9, which is reported to be upregulated in KRT1 knock-out mice alongside IL18 (Roth et al., 2012) (Supplementary Figures S4A, B). Furthermore, several genes involved in KC differentiation and epidermal keratinization were differentially regulated in AD_pep versus AD_rec (Supplementary Figure S4C). For instance, KRT5, KRT6A/B/C, KRT14, and KRT16 gene expression was upregulated, whereas KRT1, KRT10, and KRT15 expression was downregulated. KRT6 and KRT16 are involved in danger sensing via DAMPs and their expression levels have been shown to be elevated in stressed KC during wound healing and chronic skin inflammation (DePianto and Coulombe, 2004; Hobbs et al., 2012; Rotty and Coulombe, 2012; Lessard et al., 2013). KRT15, in contrast, is expressed in the hair bulge and in undifferentiated KCs of the basal layer (Cheng et al., 2018; Cohen et al., 2022). Hence, our data suggest that Der p 2 peptides induce perturbations in KC differentiation and keratinization in basal, suprabasal, and hair follicle KCs.

### 3.6 Der p 2-derived peptides upregulate the expression of mitotic and cell cycle progression genes in AD patients

**FIGURE 5:**
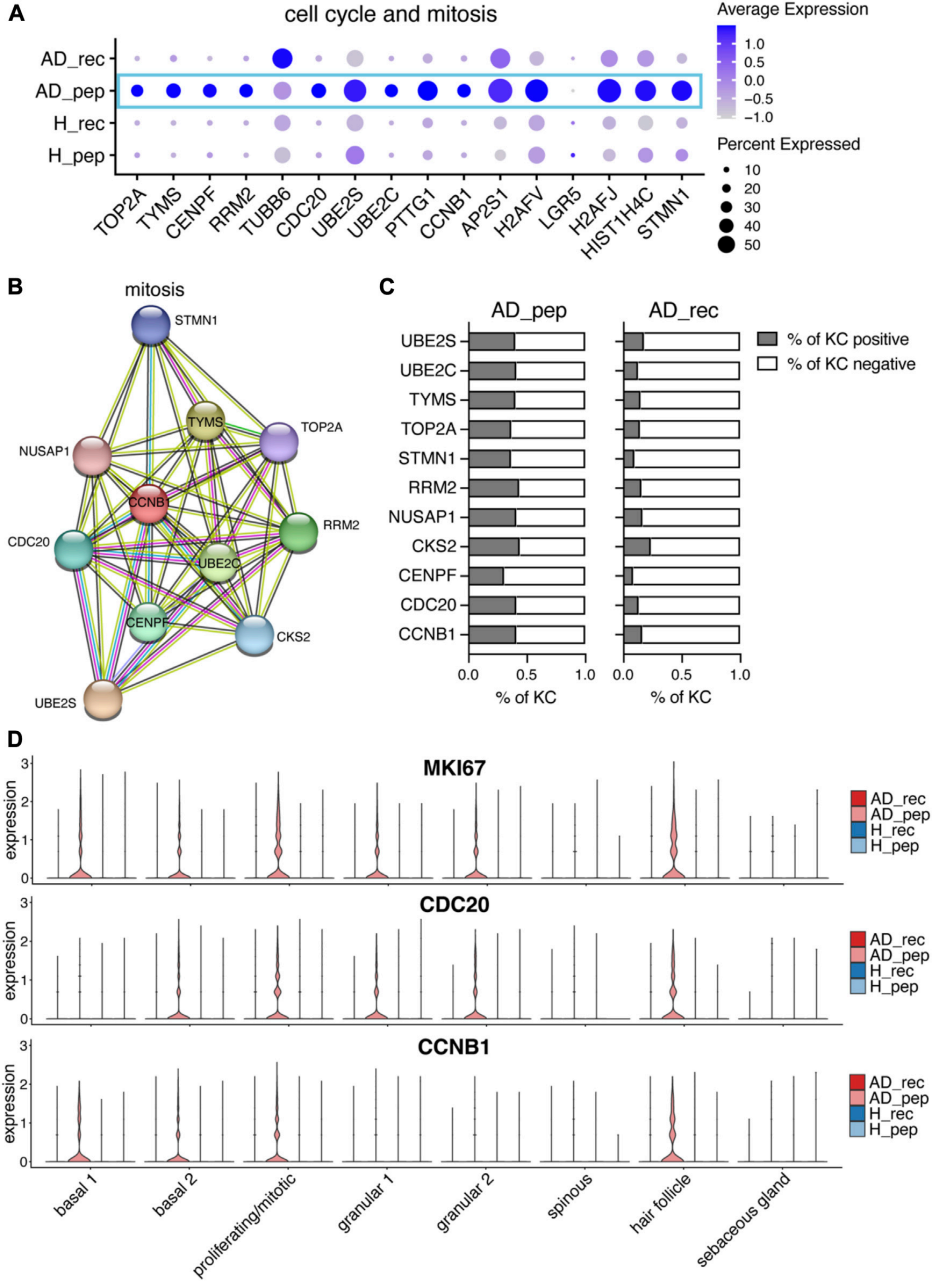
Der p 2 peptides upregulate KC hyperproliferation in AD. **(A)** Bubble plot showing the expression of cell cycle and mitotic genes in KC from skin exposed to Der p 2 protein (AD_rec and H_rec) and Der p 2 peptides (AD_pep and H_pep). **(B)** Functional enrichment analysis identified mitotic genes enriched in AD skin exposed to Der p 2 peptides. Genes identified by differential gene expression between AD_pep and AD_rec were further analyzed using the STRING network database. Identified genes are visualized by circles and their predicted associations with lines. **(C)** Percentage of KC-expressing genes identified with STRING is shown in **(B)** for AD skin exposed to Der p 2 peptides (pep) and recombinant protein (rec). **(D)** Violin plots show the average gene expression in KC clusters from AD_rec (dark pink), AD_pep (light pink), H_rec (dark blue), and H_pep (light blue) skin samples (*n* = 4 per group). AD, atopic dermatitis; H, healthy; KC, keratinocyte; rec, recombinant; pep, peptide.

Overexpression of alarmin IL18 can promote hallmark features of AD, such as type 2 skin inflammation and IL4- and IL13-induced epidermal hyperplasia (Leung et al., 2020; Beck et al., 2022). As hyperplasia is the result of increased cell proliferation, we investigated the expression of cell cycle and mitotic genes in KCs from AD_rec, AD_pep, H_rec, and H_pep samples (Figure 5A). Interestingly, a plethora of cell cycle and mitotic genes were upregulated in KCs from AD patients exposed to Der p 2 pep (Figure 5A, blue rectangle), such as DNA topoisomerase 2 (TOP2A) and centromere protein F (CENPF), which are important for chromosome segregation in mitosis; cytoskeletal genes such as tubulin beta (TUBB2); microtubule-stabilizing NUSAP1 and microtubule-destabilizing stathmin (STMN1); the cyclin-dependent kinase CKS2; and CDC20, UBE2C, and UBE2S, which encode for anaphase-promoting complex/cyclosome-regulating genes. When we analyzed genes that were specifically upregulated in AD_pep using STRING, we identified an enrichment of genes in the GO biological process pathway “mitosis” (Figure 5B) and found that a higher percentage of KCs from AD_pep expressed mitosis relevant genes compared to AD_rec (Figure 5C). This suggests that Der p 2-derived peptides can activate KC hyper-proliferation and thereby increase epidermal hyperplasia. Further investigation of genes reportedly upregulated in hyperplasia (Beck et al., 2022) revealed that Ki-67 (MKI67) expression is increased upon Der p 2 pep exposure in KC clusters basal 1 and basal 2, mitotic and hair follicle KC, and also in the suprabasal KC clusters granular 1 and granular 2 but not in spinous and sebaceous gland KCs (Figure 5D, upper panel). We found a similar expression pattern for CDC20 and the G2/M-specific cell cycle gene *CCNB1*, which both showed increased expression for AD_pep in basal, mitotic, and hair follicle KCs. Similar to MKI67, CDC20 was upregulated in granular KCs as well (Figure 5D, middle and bottom panel). Our data reveal the specific induction of hyperplasia-related genes in KC through Der p 2-derived peptides that were applied to non-inflamed skin of AD patients.

## 4 Discussion

The epidermal barrier plays a crucial role in preventing the entry of microbes, chemical irritants, and allergens into the skin. To date, there is limited information available on how allergens affect human tissue-resident skin cells at the single-cell level in terms of gene expression. To address this, we conducted patch tests on the non-lesional skin of patients with AD and healthy participants using the HDM allergen Der p 2. Although Der p 2 itself does not possess proteolytic activity like Der p 1, proteases present on the skin or in HDM feces can break down Der p 2 into smaller fragments. Therefore, we chose to compare gene expression responses to both Der p 2 protein and a mix of Der p 2 peptides in KCs from AD patients and healthy individuals using single-cell RNA sequencing technology.

Our KC cluster analysis of allergen-exposed skin confirmed the presence of all major KC subsets reported for adult skin (Cheng et al., 2018; Reynolds et al., 2021) (Figure 1). However, we could not identify a decrease in the percentage of spinous KCs in AD compared to healthy individuals, as previously suggested (Leung et al., 2020). Overall, our data revealed a clear AD-specific signature in KC from patients compared to healthy participants, confirming that skin cell differentiation is disrupted even in the absence of severe skin inflammation, as described previously (Suárez-Fariñas et al., 2011). This finding was confirmed by the global comparison of gene expression data from AD and H, where we found a significant impairment of the skin barrier in AD patients (Akdis, 2021; Mitamura et al., 2021) characterized by the downregulation of genes involved in the cell–cell junction assembly (Figures 2B, C). Consistent with this, we also observed a profound downregulation of genes regulating tight junctions, namely, CLDN1 (De Benedetto et al., 2011), PARD3 (Ali et al., 2016), and TIAM1 (Mertens et al., 2005), another hallmark of AD (Supplementary Figure S2B).

Recombinant Der p 2, but not hypoallergenic Der p 2 peptides, can be presented by IgE-facilitated mechanisms. This may explain the specific upregulation of certain immune system relevant genes by the full protein, such as SERPINB1, CALR, CCL27, and S100A7 (Figure 3). Furthermore, Der p 2 protein has been shown to activate the innate immune system by mimicking MD2-related lipid recognition domains (Trompette et al., 2009; Kaplan et al., 2012; Eyerich and Novak, 2013; Reithofer and Jahn-Schmid, 2017; Smith et al., 2017). The protease inhibitor SERPINB3 has been found to be upregulated in the lesional skin of AD patients, and its expression is induced by type 2 cytokines (Mitsuishi et al., 2005). Despite low inflammation scores in our patch tests, we observed an upregulation of SERPINB3 in basal, spinous, granular, and hair follicle KCs of AD skin (Figure 3). Previous studies in mice have shown that SERPINB3 likely contributes to early skin inflammation in AD (Sivaprasad et al., 2015). Interestingly, exposure to Der p 2 recombinant proteins further increased SERPINB3 expression, particularly in spinous KC. This suggests that even without proteolytic cleavage of tight junctions, allergens can overcome the skin barrier in AD, induce inflammatory pathways, and activate an early inflammatory gene expression signature in KC. This fact can be attributed to the preexisting barrier dysfunction in AD, allowing proteins such as allergens to penetrate deep into the skin.

Increased expression of S100A7 in suprabasal KCs has been reported after barrier disruption by tape stripping of the healthy skin (Gläser et al., 2009). Analysis of S100A7 expression in our samples using feature plots (data not shown) and violin plots (Figure 3B) revealed that S100A7 was not uniformly expressed in KCs. In particular, granular and hair follicle KCs illustrated a bimodal expression pattern of S100A7. We speculate that certain KC cells that reside on the outer surface of the skin may be more prone to experiencing environmental impacts and consequently respond with similar gene expression patterns compared to KC in deeper epidermal layers. Further studies will show whether cells expressing high levels of S100A7 localize to the same niche within the skin.

Comparing gene expression changes induced by Der p 2 protein and Der p 2 peptides, we found a marked reduction in the expression of genes associated with hemidesmosomes and focal adhesions in KCs from AD skin treated with Der p 2 peptides (Figures 4A, B). In a mouse model, the disruption of the anchorage of intestinal epithelial cells to the BM through loss of hemidesmosomes led to caspase-1 activation and increased IL18 secretion (De Arcangelis et al., 2017). Similarly, exposure of skin in AD patients to Der p 2 peptides reduced the expression of hemidesmosome genes and increased PYCARD and IL18 expression in proliferating/mitotic, spinous, and granular KCs (Figures 4D, E). This suggests a similar activation pathway in skin KC, which may be further enhanced by the disruption of ECM organization (Supplementary Figure S3B) (Bhattacharjee et al., 2019; Pfisterer et al., 2021). Another study in mice demonstrated that the downregulation of KRT1, along with increased IL18, S100A8, and S100A9, caused barrier defects in the skin (Roth et al., 2012). In AD patients, HDM extracts have been shown to increase the Th2 signature and upregulate S100A7 and S100A8 in the skin (Malik et al., 2017). Our *in vitro* barrier disruption experiment suggests that Der p 2 peptides can directly affect tight junctions by downregulating claudin 1 expression in the absence of other cells. Although the mechanism remains elusive, our findings suggest that Der p 2 peptides in HDM feces may play a role in driving this switch.

KRT1 expression was particularly downregulated in KC clusters, showing the upregulation of PYCARD and IL18 upon skin exposure to Der p 2 peptides (Figure 4E). *In vitro* studies with isolated KC have shown that Der p 1, but not Der p 2, induced the assembly of the NLRP3 inflammasome, leading to caspase-1 activation, and IL-1β and IL18 secretion (Dai et al., 2011). In contrast, we observed a clear upregulation of PYCARD and IL18 by Der p 2 peptides and a less pronounced effect by the full protein. This discrepancy may be due to the higher sensitivity of scRNA-seq, which can detect subtle changes in gene expression on the single-cell level. Moreover, our data suggest that the effect seen for recombinant Der p 2 protein may depend on IgE-dependent immune system activation, whereas Der p 2 peptides may directly affect KCs in the absence of IgE reactivity. High levels of IL4 and IL13 have been reported to reduce KRT1 expression in the lesional skin of AD patients (Beck et al., 2022). Interestingly, we found in our study that KRT1 expression was particularly downregulated upon AD skin exposure to Der p 2 peptides (Supplementary Figure S4C). In contrast, KRT6A/B/C and KRT16 were upregulated, which, along with KRT1 downregulation, have been implicated in causing barrier disruptions (Hobbs et al., 2012).

IL18, a proinflammatory pleiotropic cytokine, can modulate both the innate and the adaptive immune systems. When IL18 was identified and named interferon-gamma-inducing factor, it was found to induce type 1 cytokine production in the presence of IL12. However, in the absence of IL12, IL18 enhances type 2 cytokine production by helper T cells, mast cells, and basophils (Yoshimoto et al., 1999). Our data suggest that Der p 2 peptides induced IL18 and may skew the immune system toward an elevated type 2 response as we detected IL13 but not IL12 in our dataset comprising all skin cells (data not shown). As IL18 can potentially regulate both type 1 and type 2 cytokine productions, it makes it a prominent candidate to control the switch between acute and chronic AD, which are characterized by type 2 and type 1 cytokines, respectively (Langan et al., 2020).

IL4 and IL13 promote epithelial cell proliferation and hyperplasia, which are specifically triggered during AD initiation and acute lesions (Gittler et al., 2012; Beck et al., 2022). We examined the expression of cell cycle and mitosis genes and found a specific upregulation of mitosis genes in KC from AD skin exposed to Der p 2 peptides (Figure 4). Further analysis of upregulated genes in Der p 2 peptide-treated skin revealed an enrichment of genes involved in the mitosis pathway and a higher percentage of KC expressing mitosis genes in AD_pep compared to AD_rec. Known markers for hyperplasia, such as MKI67, S100A8, S100A9 (Beck et al., 2022), and K16, were all upregulated. Interestingly, MKI67 was upregulated in most KC clusters, indicating a proliferative response to Der p 2 peptides in both basal and suprabasal KC, with the exception of spinous and sebaceous KCs.

In conclusion, our findings suggest that Der p 2 peptides may contribute to initiating major hallmark features of AD, including allergic inflammation, barrier disruption, and hyperplasia. Future studies will reveal that whether the observed net effect on KC is due to immune cell activation or a direct effect of Der p 2 peptides on KC in AD.

## Data Availability

The analyzed datasets for this study can be found in Supplementary Tables S6–S14. The raw count matrix (https://doi.org/10.6084/m9.figshare.23898333.v1) and phenotype file (https://doi.org/10.6084/m9.figshare.23898330.v1) presented in this study were deposited in Figshare.
