# Supplementary material for: Non-IgE-reactive allergen peptides deteriorate the skin barrier in house dust mite-sensitized atopic dermatitis patients
Source: Front Cell Dev Biol. 2023 Aug 22;11:1240289. doi: 10.3389/fcell.2023.1240289 (PMC10478000; doi:10.3389/fcell.2023.1240289)
Supplement: Supplementary file 1 [file DataSheet1.pdf]

Supplementary Material

Non-IgE-reactive allergen peptides deteriorate the skin barrier in house dust mite-sensitized atopic dermatitis patients

Karin Pfisterer[1*], Matthias Wielscher[1], David Samardzic[1], Pauline Weinzettl[1], Dörte Symmank[1], Lisa E Shaw[1], Raffaela Campana[2], Huey-Jy Huang[2,3], Matthias Farlik[1], Christine Bangert[1], Susanne Vrtala[2], Rudolf Valenta[2], Wolfgang Weninger[1*]

*** Correspondence:**

Karin Pfisterer (karin.pfisterer@meduniwien.ac.at)

Wolfgang Weninger (w.weninger@meduniwien.ac.at)

1 Supplementary Figures

Supplementary Figure 1

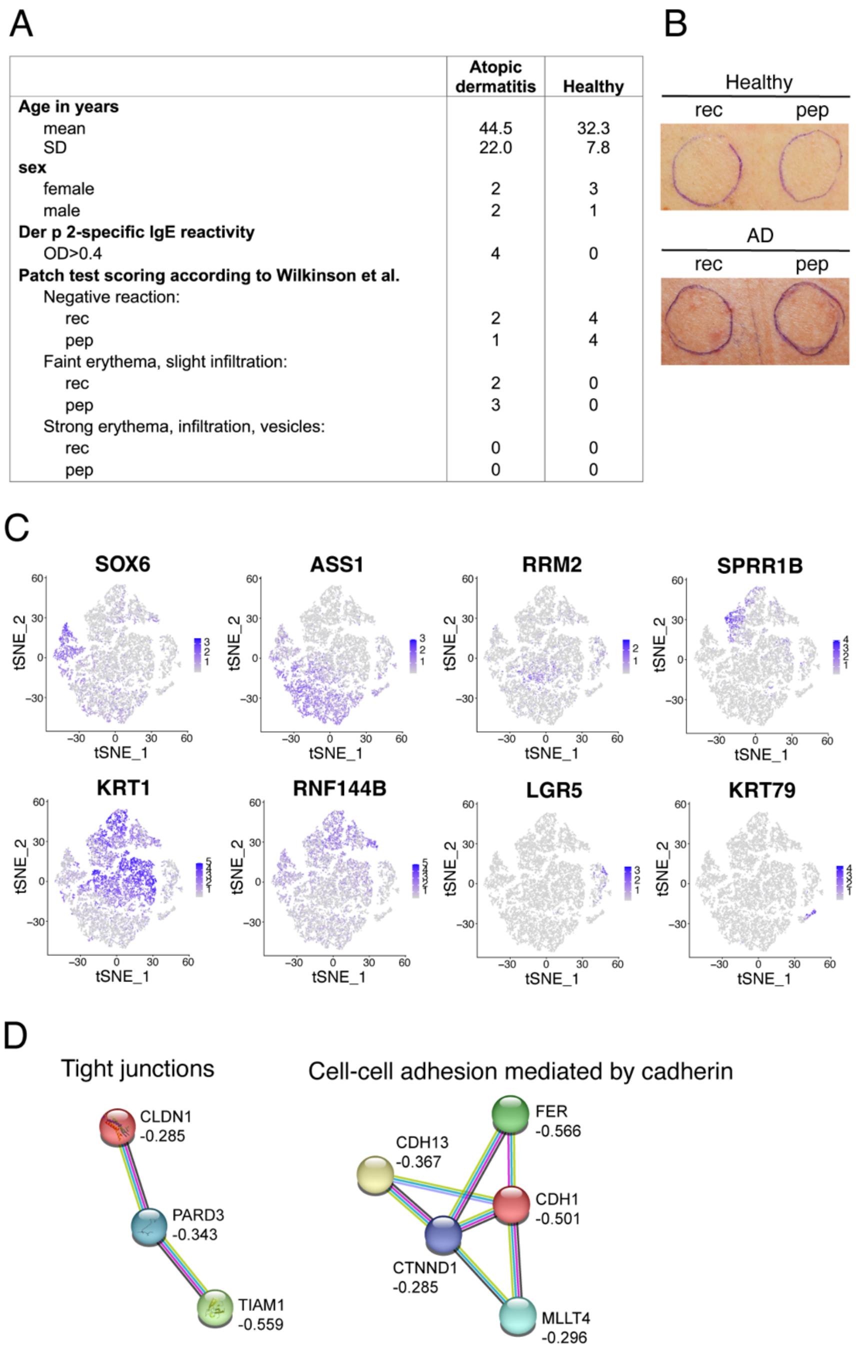

	Atopic dermatitis	Healthy
**Age in years**		
mean	44.5	32.3
SD	22.0	7.8
**sex**		
female	2	3
male	2	1
**Der p 2-specific IgE reactivity**		
OD>0.4	4	0
**Patch test scoring according to Wilkinson et al.**		
Negative reaction:		
rec	2	4
pep	1	4
Faint erythema, slight infiltration:		
rec	2	0
pep	3	0
Strong erythema, infiltration, vesicles:		
rec	0	0
pep	0	0

Supplementary Figure 1. Description of identified KC subsets and barrier function status. **A)** Demographic table summarizing data of study participants and patch test scorings. **B)** Representative images of Der p 2 rec and Der p 2 pep treated healthy and AD skin. **C)** Shown are

tSNE plots highlighting cell cluster specific gene expression. KC clusters from the merged dataset comprising all AD and H biopsies (16 biopsies in total) are shown in grey and gene expression values in blue (low expression, light blue; high expression, dark blue). Upper panel (from left to right): basal 1, basal 2, proliferating/mitotic, spinous; lower panel (from left to right): granular 1, granular 2, hair follicle, sebaceous gland KC. **B)** Functional enrichment analysis with STRING using downregulated genes in AD compared to H identified defects in tight junctions (left) and cell-cell adhesions mediated by cadherin (right), which is typical for adherens junctions. Shown is the network view of genes (circles), their predicted associations (lines) and the average log2 fold change values (see Suppl. Table 6). AD, atopic dermatitis; H, healthy; rec, recombinant; pep, peptide; tSNE, t Distributed Stochastic Neighbor Embedding.

Supplementary Figure 2

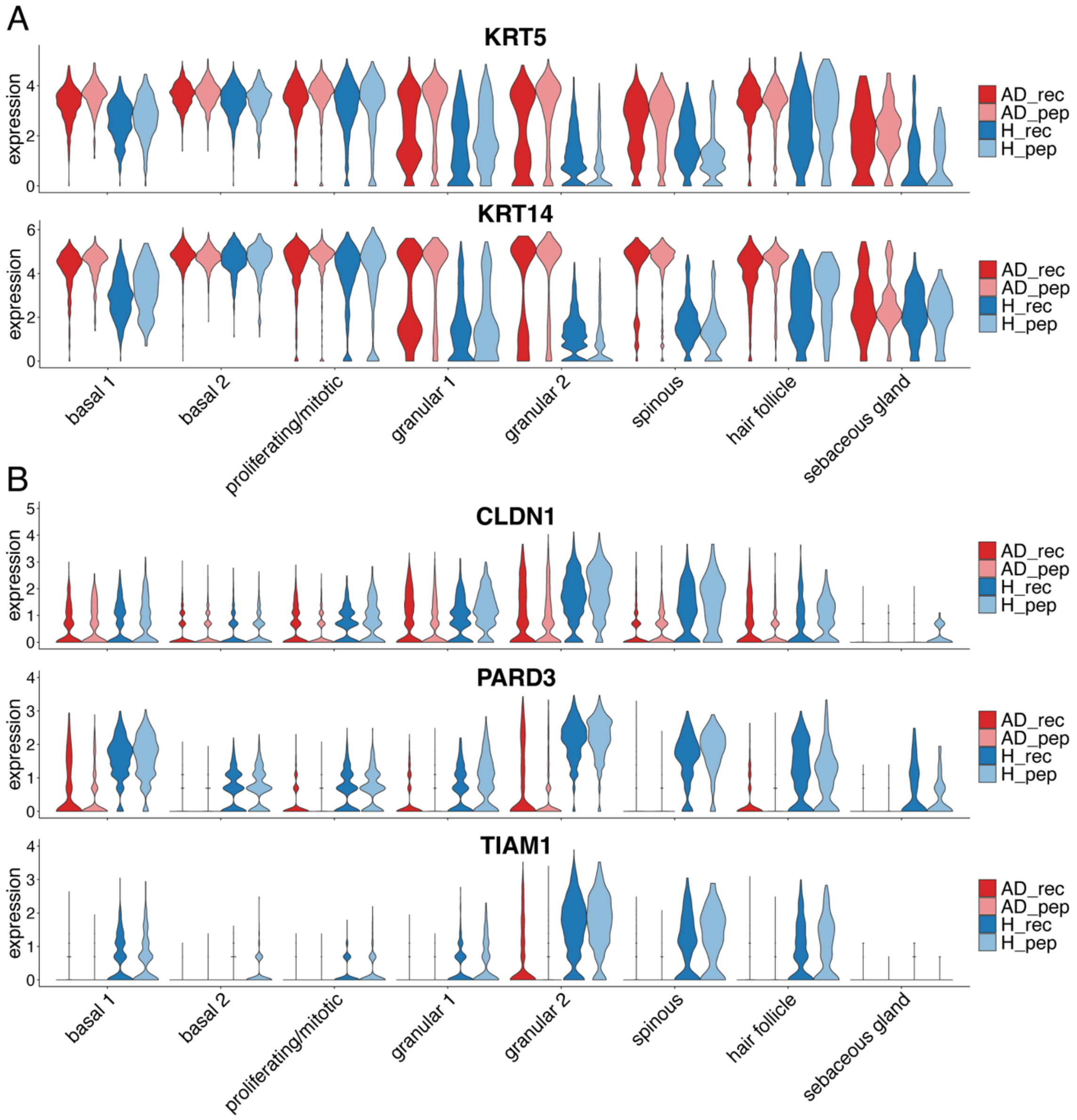

Supplementary Figure 2. Der p 2 affected KC differentiation and tight junction formation. (A-B) Violin plots show the average gene expression in KC clusters from AD_rec (dark pink), AD_pep (light pink), H_rec (dark blue) and H_pep (light blue) skin samples (n=4) for the keratin genes KRT5 and KRT14 (**A**) and for the tight junction genes CLDN1, PARD3, TIAM1 (**B**). Interestingly KRT5 and KRT14 was not efficiently downregulated in suprabasal KC in AD skin samples. AD, atopic dermatitis; H, healthy; KC, keratinocyte; pep, peptide; rec, recombinant protein.

Supplementary Figure 3

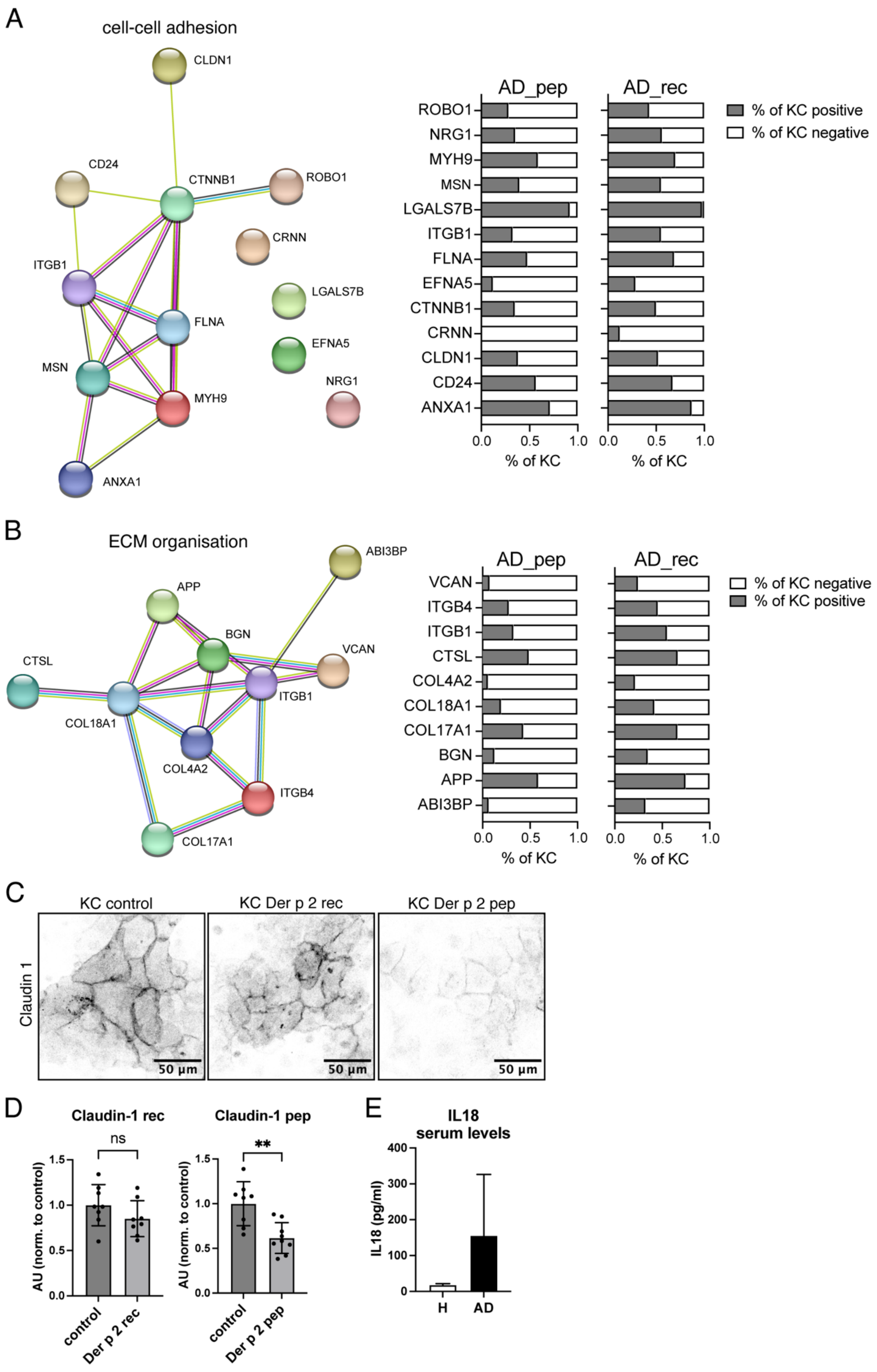

Supplementary Figure 3. Der p 2 peptides reduced the expression of cell-cell adhesion and ECM organization genes that are essential to maintain the skin barrier.

(A-B) Differential gene expression analysis of AD_pep and AD_rec resulted in a list of 98 downregulated genes (Supplementary table 10), which were further analyzed using the functional enrichment analysis tool STRING. Shown are the interaction networks and predicted functional associations for cell-cell adhesion (**A**) and ECM organization (**B**) genes identified in GO Biological Process (n=4). Right: Bar graphs depict the percentage of KC expressing the respective genes in AD_pep and AD_rec samples. AD, atopic dermatitis; ECM, extracellular matrix; KC, keratinocyte; pep, peptide; rec, recombinant protein. **C)** Claudin 1 expression was downregulated in differentiated KC upon exposure to Der p 2 pep. Shown are representative images control, Der p 2 rec and Der p 2 pep treated differentiated KC stained for claudin 1 and imaged with a confocal microscope. Scale bar = 50 μm. **D)** Quantification of images shown in C). Shown is the statistical analysis of a representative experiment. ** $p<0.002$. E) IL18 serum levels in AD and H participants were measured by ELISA. n=4 for AD and H.

Supplementary Figure 4

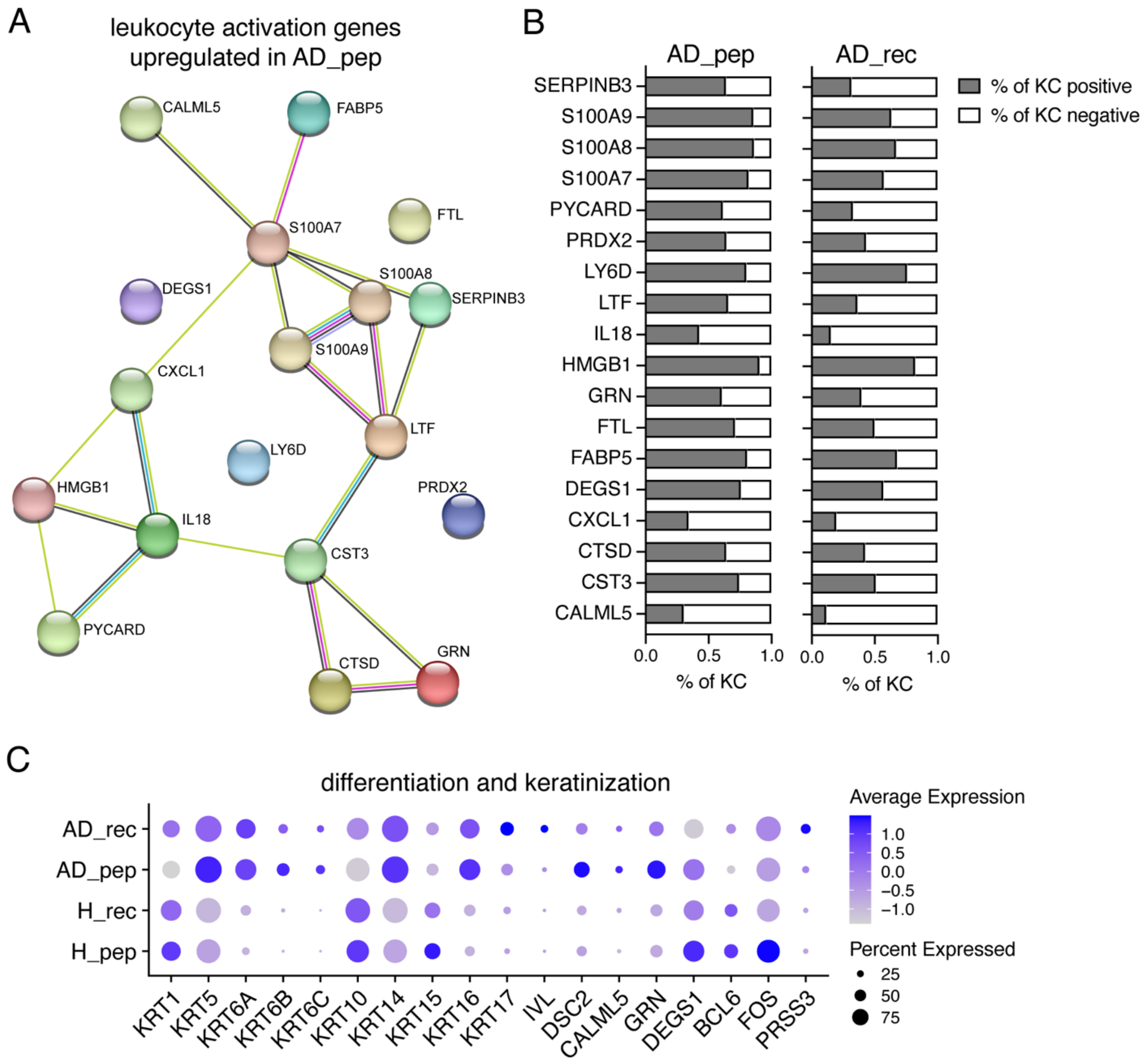

Supplementary Figure 4. Der p 2 pep upregulated inflammation associated genes and altered KC differentiation.

(**A**) 197 upregulated genes from the comparison AD_pep versus AD_rec (Supplementary table 10) were further analyzed using the STRING database to identify functional enrichments in the gene set. The GO Biological Process pathway leukocyte activation was identified. Shown are the interaction networks and predicted functional associations for AD_pep. (**B**) The bar graph shows the percentage of KC expressing the respective genes from (**A**) in AD_pep and AD_rec samples. (**C**) Bubble plot showing the expression of differentiation and keratinization associated genes in KC from skin exposed to recombinant Der p 2 protein (AD_rec, H_rec) and Der p 2 peptides (AD_pep, H_pep).

Many KRT genes were differentially regulated in AD_pep. AD, atopic dermatitis; H, healthy; KC, keratinocyte; pep, peptide; rec, recombinant protein.

2 Supplementary Tables

Supplementary Table 1. Shown is the quality control (QC) overview including threshold and PC settings, number of excluded cells per sample, doublet analysis, number of cells and genes post QC, average UMIs per gene and per cell, and average genes per cell. QC was undertaken for each sample separately (cells from each biopsy), the quality was assessed and only high-quality data were merged for further analysis.

Supplementary Table 2. Shown is the cluster overview of the merged dataset. After individual QC, all samples were merged and clusters defined. The table includes the number of cells per cluster, average UMIs and mean features per cluster.

Supplementary Table 3. The table shows the number of cells in each cluster for the sample groups AD_pep, AD_rec, H_pep and H_rec including P values from CHI Square test (Bonferroni corrected).

Supplementary Table 4. Shown are the genes that differed between clusters. Depicted are comparisons between all clusters and the respective average log2 fold change (avg_log2FC) and P values from Wilcox rank sum test.

Supplementary Table 5. Gene set enrichment analysis to identify genes that are differentially expressed between clusters. Shown are the top five overlapping pathways from the KEGG_2019_Human database, the ARCHS4_Tissues database and the MSigDB_Hallmark_2020 database, the overlap, P value, adjusted P value and the combined score value.

Supplementary Table 6. Shown are differential genes from the global comparison of AD and H samples, including the comparisons AD_pep versus H_pep, and AD_rec versus H_rec. Listed are the gene IDs, the P value, average log2 fold change values, the percentage of KC expressing the gene in the case cluster (pct.1) and the control cluster (pct.2), the adjusted P value, the cluster sizes and total UMI counts for case and control, and the AUC.

Supplementary Table 7. Shown are differential genes from the comparison of AD_pep versus H_pep for each cluster. Listed are the gene IDs, the P value, average log2 fold change values, the

percentage of KC expressing the gene in the case cluster (pct.1) and the control cluster (pct.2), the adjusted P value, the cluster sizes and total UMI counts for case and control, and the AUC.

Supplementary Table 8. Shown are differential genes from the comparison of AD_rec versus H_rec for each cluster. Listed are the gene IDs, the P value, average log2 fold change values, the percentage of KC expressing the gene in the case cluster (pct.1) and the control cluster (pct.2), the adjusted P value, the cluster sizes and total UMI counts for case and control, and the AUC.

Supplementary Table 9. Shown are differential genes from the global comparison of AD_rec versus AD_pep. Listed are the gene IDs, the P value, average log2 fold change values, the percentage of KC expressing the gene in the case cluster (pct.1) and the control cluster (pct.2), the adjusted P value, the cluster sizes and total UMI counts for case and control, and the AUC. Please note that the comparison H_rec versus H_pep did not result in any differential genes.

Supplementary Table 10. Shown are differential genes from the comparison of AD_rec versus AD_pep for each cluster. Listed are the gene IDs, the P value, average log2 fold change values, the percentage of KC expressing the gene in the case cluster (pct.1) and the control cluster (pct.2), the adjusted P value, the cluster sizes and total UMI counts for case and control, and the AUC.

Supplementary Table 11. Listed are genes that were driving the trajectories for the merged dataset including all healthy and AD skin samples (Fig. 2A), the clusters expressing these genes and the fitted p value kinetics.

Supplementary Table 12. Listed are genes that were driving the trajectories for AD skin samples (Fig. 2A, left plot), the clusters expressing these genes and the fitted p value kinetics.

Supplementary Table 13. Listed are genes that were driving the trajectories for healthy skin samples (Fig. 2A, right plot), the clusters expressing these genes and the fitted p value kinetics.

Supplementary Table 14. Genes from the global differential comparison of AD_pep and AD_rec (Suppl. Table 9) were further analyzed using the STRING tool to identify gene set enrichments in the GO Biological Process database. Shown are genes that were enriched in cell cycle and mitosis pathways.
